# Tuina Alleviates Neuropathic Pain in CCI Rats by Regulating the TRPV4-CaMKII Signaling Pathway in Dorsal Root Ganglion

**DOI:** 10.1155/prm/3697374

**Published:** 2025-08-06

**Authors:** Rentuya Na, Yue Xu, Tianyuan Yu, Yingqi Zhang, Jiawang Yan, Hongzheng Zhang, Hanyu Zhang, Jiawei Sun, Jiayue Liu

**Affiliations:** School of Acupuncture-Moxibustion and Tuina, Beijing University of Chinese Medicine, Beijing 102446, China

**Keywords:** dorsal root ganglion, neuropathic pain, peripheral sensitization, TRPV4, Tuina

## Abstract

**Background:** Peripheral sensitization mediated by the Transient Receptor Potential Vanilloid 4-Calcium/calmodulin-dependent protein kinase II (TRPV4-CaMKII) signaling pathway plays a fundamental role in the generation and maintenance of neuropathic pain (NP). Tuina, a safe and effective therapy in traditional Chinese medicine, shows analgesic effects; however, the underlying mechanisms remain unclear. We aimed to investigate whether Tuina alleviates pain by modulating the TRPV4-CaMKII/CREB/NLRP3 signaling pathway.

**Methods:** The Chronic Constriction Injury (CCI) model of the sciatic nerve was used to simulate clinical NP. Tuina was applied to the Yinmen (BL37), Yanglingquan (GB34), and Chengshan (BL57) acupoints once daily for 14 days. Mechanical Withdrawal Threshold (MWT) and Thermal Withdrawal Latency (TWL) were assessed to evaluate the analgesic effect of Tuina. Its protective effects on dorsal root ganglion (DRG) neurons were evaluated using Nissl staining. The whole-cell patch clamp technique recorded excitability changes in DRG neurons and assess the effects of Tuina on peripheral sensitization. Western blot (WB), immunofluorescence (IF), and enzyme-linked immunosorbent assay (ELISA) helped detect changes in the TRPV4-CaMKII/CREB/NLRP3 pathway and expression of inflammation-related cytokines in DRG neurons.

**Results:** Tuina significantly alleviated mechanical allodynia and thermal hyperalgesia in CCI rats and exerted a protective effect on DRG neurons. Patch clamp recordings showed that Tuina inhibited hyperexcitability in DRG neurons. Mechanistically, Tuina downregulated the expression of the TRPV4-CaMKII/CREB/NLRP3 signaling pathway and reduced the secretion of TNF-α, IL-1β, and IL-18.

**Conclusion:** The analgesic effect of Tuina in CCI rats is associated with reduced peripheral sensitization via modulation of the TRPV4-calcium signaling cascade.

## 1. Introduction

Neuropathic pain (NP) is defined as pain arising directly from a lesion or disease affecting the somatosensory nervous system [1]. NP poses a significant challenge to public health and quality of life, affecting approximately 6.9%–10% of the global population [2]. Pharmacological treatment remains the primary approach for symptom relief; however, limitations such as drug resistance, dependence, and adverse effects hinder the prolonged use of monotherapies [3]. Consequently, identifying effective therapies with minimal side effects remains an urgent clinical priority.

Pain typically originates from the activation and sensitization of peripheral sensory neurons—a process known as peripheral sensitization. These neurons detect nociceptive signals and transmit them to the spinal cord and supraspinal structures, including the thalamus, somatosensory cortex, and anterior cingulate cortex, ultimately contributing to central sensitization [4]. Thus, inhibiting peripheral sensitization may help attenuate or delay subsequent central nervous system events [5, 6].

Peripheral sensitization relies on the activation and upregulation of ion channels in neurons. The Transient Receptor Potential Vanilloid 4 (TRPV4) receptor channel is a nonselective cation channel expressed in the dorsal root ganglion (DRG) [7, 8]. Per previous studies, TRPV4 activation facilitates calcium influx, initiates action potentials, and markedly increases the excitability of DRG neurons [9, 10]. Conversely, inhibition of TRPV4 ion channels reduces the hyperexcitability of peripheral fibers following peripheral nerve injury, thereby significantly improving NP-related behaviors [10–13]. These findings suggest that TRPV4 is a promising therapeutic target for NP.

Calcium/calmodulin-dependent protein kinase II (CaMKII) is a widely expressed serine/threonine kinase in the nervous system, and its aberrant activation has been strongly implicated in the pathogenesis of NP [14]. Evidence suggests that TRPV4-mediated calcium influx leads to intracellular calcium overload, which subsequently activates CaMKII via phosphorylation [15, 16]. Activated CaMKII, in turn, phosphorylates cAMP response element-binding protein (CREB) and regulates the transcription of multiple pain-related genes, contributing to the development and maintenance of peripheral sensitization [17–19]. Therefore, targeting the TRPV4-CaMKII signaling pathway and its downstream cascades holds significant promise for the treatment and prevention of NP.

Tuina is a nonpharmacological therapy rooted in traditional Chinese medicine and is classified as a form of physical therapy. By applying various manipulative techniques, such as pressing and kneading the body surface, Tuina aims to warm and unblock meridians, promote the circulation of qi and blood, and thereby alleviate pain and numbness [20, 21]. In China, Tuina is widely used in the clinical management of various pain conditions, demonstrating significant therapeutic efficacy, minimal adverse effects, and other additional benefits [22–24]. Although, per recent studies, Tuina produces therapeutic effects in NP, the underlying mechanisms of its analgesic action, particularly regarding its influence on peripheral sensitization, remain incompletely understood. Our previous RNA-sequencing (RNA-seq) analysis suggested that the therapeutic effects of Tuina are associated with the modulation of TRP channels and CaMKII signaling pathways [25, 26]. Based on these findings, we further investigated whether Tuina mitigates peripheral sensitization and alleviates NP by modulating TRPV4 ion channels and their downstream signaling cascades in DRG neurons.

In this study, we used behavioral tests and Nissl staining to evaluate the cumulative analgesic effects of Tuina. The patch-clamp technique was used to assess the effects of Tuina on the excitability of DRG neurons. Immunofluorescence, Western blotting, and enzyme-linked immunosorbent assay (ELISA) were performed to measure the expression of the TRPV4-CaMKII/CREB/NLRP3 signaling pathway and pro-inflammatory cytokines TNF-α, IL-1β, and IL-18 in DRG tissue. In conclusion, we investigated whether Tuina exerts analgesic effects by reducing peripheral sensitization through regulation of the TRPV4 ion channels and their associated signaling cascades in the DRG.

## 2. Materials and Methods

### 2.1. Animals

Male Sprague-Dawley rats (200 ± 10 g) were obtained from SPF (Beijing) Biotechnology Co., Ltd. (Experimental animal license: SCXK (JING) 2024-0001). The rats were housed in the Animal Experiment Center of Beijing University of Chinese Medicine, with five rats per cage. Environmental conditions were maintained at a temperature of 25 ± 2°C with a 12-h light/dark cycle. A total of 50 rats were enrolled in this study. Experiment 1 included 18 rats, which were randomly assigned to the Sham group, the CCI group, and the Tuina group, with six rats in each group. To further elucidate the role of the TRPV4 ion channel in Tuina-induced analgesia, Experiment 2 was conducted. Experiment 2 included 32 rats, which were randomly assigned to the Sham group, the CCI group, the Tuina group, and the Tuina + GSK group, with eight rats in each group.

Rats were acclimatized to laboratory conditions for 1 week prior to the start of experiments. All experimental procedures were approved by the Ethics Committee of Beijing University of Chinese Medicine (License No. BUCM-2024090203-3127) and conducted in accordance with the National Institutes of Health Guidelines for the Care and Use of Laboratory Animals.

### 2.2. Modeling Surgery

The CCI and Tuina groups underwent CCI modeling surgery [27].

In brief, fasting was fasted for 12 h before surgery. The rats were fixed in the prone position, and gas anesthesia was performed with isoflurane. The right sciatic nerve was exposed. Four 4–0 loose chromic gut ligatures were loosely placed at the proximal end of the sciatic nerve bifurcation prior to wound closure. The Sham group underwent the same surgical procedure as the CCI group, but only the sciatic nerve was exposed without ligation. The patients were fasted for 12 h after surgery, and the wound condition and foot changes were observed regularly during the experiment.

### 2.3. Intervention Methods

**Figure 1:**
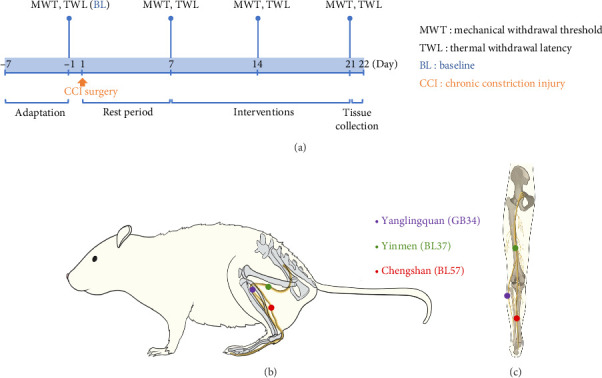
Experimental timeline and Tuina acupoints. (a) Timeline of the experiment and (b and c) the relative positions of the Yanglingquan (GB34), Yinmen (BL37), and Chengshan (BL57) acupoints and their corresponding acupoints in humans.

All interventions began on day 7 post-CCI surgery, once the model was successfully established and stabilized (Figure 1(a)).

Tuina group: received treatment involving three manipulations and stimulation of three acupoints. The manipulations consisted of point-pressing, plucking, and kneading. The selected acupoints were Yanglingquan (GB34), Yinmen (BL37), and Chengshan (BL57) (Figures 1(b) and 1(c)). A self-developed Tuina manipulation instrument (Chinese invention patent number: ZL202320511277.5) was utilized to ensure precise and quantitative intervention for each acupoint. Parameters were set as follows: the intensity was 4 N, the frequency was 60 times per minute, and each manipulation and acupoint was intervened for 1 min. The total intervention time was 9 min. The intervention was administered once daily for a period of 14 days. Sham and CCI group: grip restraint was performed for 9 min per day.

### 2.4. Intraperitoneal Injection

To investigate the relationship between the analgesic effect of Tuina and TRPV4 ion channels, GSK1016790A (10 μg/kg, MedChemExpress, United States) was administered intraperitoneally 30 min prior to the Tuina intervention [28]. GSK1016790A is a potent agonist that has been extensively used to investigate the in vivo pharmacology of TRPV4 activation.

### 2.5. Behavioral Tests

The MWT was used to evaluate mechanical allodynia, while the TWL was used to assess thermal hyperalgesia. The MWT and TWL assessments were comprehensively detailed in our prior research [25].

### 2.6. Tissue Selection

After 14 days of interventions, the rats were euthanized under anesthesia, and right L4-L6 DRGs were rapidly isolated for whole-cell patch-clamp recordings. The right L4-L6 DRGs were collected and stored at −80°C for subsequent ELISA and Western blot (WB) analyses. Following perfusion with saline and 4% paraformaldehyde, the right L4-L6 DRGs were fixed in paraformaldehyde for 24 h. The tissues were subsequently processed for cryosectioning or paraffin embedding and sectioned for Nissl staining and immunofluorescence.

### 2.7. Whole-Cell Patch Clamp

Preparation of DRG Neurons: The right L4-L6 DRG neurons were promptly isolated and treated with 1 mL of D-Hanks' solution containing 10 μL collagenase for a duration of 5–10 min. The digestion mixture was removed, and the neurons were exposed to 1 mL of 0.25% EDTA-trypsin solution for 15–20 min at 37°C. To halt the digestion process, DMEM enriched with 10% fetal bovine serum (FBS) was added. Next, the cell suspension underwent centrifugation at 1000 rpm for 3–5 min, after which the cell pellet was reconstituted in 1 mL of neurobasal medium. The isolated neurons were maintained in a culture medium that included 2% B27 supplement, 50 ng/mL nerve growth factor (NGF), 2 mmol/L L-glutamine, and penicillin/streptomycin. To improve neuronal purity, optimal concentrations of cytosine and uracil were incorporated into the culture medium. Single-cell suspensions were generated by gently pipetting the cells, followed by resuspension and filtration using a 40 μm cell strainer. These single-cell suspensions were subsequently transferred to cell culture dishes and incubated at 37°C in a humidified atmosphere with 5% CO_2_ for patch-clamp experiments.

Action potentials were measured from rat DRG neurons using whole-cell patch-clamp methods. The experiments were performed at room temperature (22°C–24°C) with an EPC-10 amplifier and Patch-Master software (HEKA, Freiburg). The extracellular solution composition was as follows (in mM): 137 NaCl, 4 KCl, 10 HEPES, 5 D-glucose, 1 MgCl2, and 2 CaCl2, adjusted to pH 7.4 using NaOH. For the intracellular solution, the following components were used (in mM): 135 KCl, 2 MgCl2, 10 HEPES, 1 EGTA, 0.3 NaGTP, and 4 Mg-ATP, with the pH adjusted to 7.4 using KOH. To record action potentials, cells were maintained at a holding potential of −80 mV in current clamp mode (*I* = 0). Action potentials were triggered and recorded by applying a series of square wave current steps, which ranged from −50 Pa to +240 Pa in 10 Pa increments, each lasting 500 ms.

### 2.8. WB

Western blotting was performed to quantify the protein expression levels of TRPV4, phospho-CaMKII (p-CaMKII), phospho-CREB (p-CREB), and NLRP3 in protein extracts from DRG neurons. DRG was homogenized in the protein lysate (Servicebio Technology, Wuhan, China), followed by centrifugation and collection of the supernatant. Protein samples were prepared by measuring the protein concentration using the BCA method according to the manufacturer's protocol. After blocking for 1 h, primary antibodies against TRPV4 (1:1000, Affinity, China), p-CaMKII (1:1000, Affinity, China), CaMKII (1:1000, Affinity, China), p-CREB (1:1000, Affinity, China), CREB (1:1000, Affinity, China), NLRP3 (1:1000, Servicebio, China), and β-actin (1:3000, Servicebio, China) were added and incubated overnight at 4°C. Following membrane washing, the corresponding secondary antibody (1:5000, Servicebio, China) was added, and the membranes were incubated for 1 h at room temperature. After washing, the protein gray values were quantified using ImageJ software.

### 2.9. ELISA

The levels of TNF-α, IL-1β, and IL-18 in the DRG tissue were determined using ELISA kits (Jiangsu Kete Biotechnology, China) according to the manufacturer's instructions.

### 2.10. Immunofluorescence

DRG tissue were processed, frozen, embedded, and sectioned into 8-μm slices. To improve membrane permeability, these sections were incubated in a solution containing 0.3% Triton X-100 for 30 min at ambient temperature. Next, to minimize nonspecific antibody binding, the sections were treated with a blocking solution of 5% BSA for 1 h at room temperature. Following the blocking step, the sections underwent three washes with PBS. The slides were then incubated overnight at 4°C with primary antibodies against TRPV4 (1:200, Affinity, China) for immunofluorescence labeling. After another series of three PBS washes, an Alexa 594-conjugated goat antimouse secondary antibody (1:500, Abways, China) was applied and incubated for 2 h at room temperature. Cellular nuclei were stained with DAPI. Fluorescent images were acquired using fluorescence microscopy, and ImageJ software was utilized for subsequent image analysis.

### 2.11. Nissl Staining

The DRG tissue were fixed, embedded in paraffin, and cut into 8-μm-thick sections. These sections were then stained with toluidine blue solution (Solarbio, Beijing, China) for 30 min at a temperature of 50°C. To eliminate excess dye, the sections were destained using 95% ethanol. Microscopic examinations and image captures were performed thereafter.

### 2.12. Statistical Analysis

Statistical analysis was conducted using GraphPad Prism 9.0. Data are presented as mean ± standard error of the mean (SEM). One-way analysis of variance (ANOVA) was used to compare multiple groups, followed by Tukey's post hoc test for pairwise comparisons when overall significance was achieved. Statistical significance was defined as *p* < 0.05, with highly significant differences defined as *p* < 0.01.

## 3. Results

### 3.1. Tuina Alleviates Mechanical Allodynia and Thermal Hyperalgesia in CCI Rats

**Figure 2:**
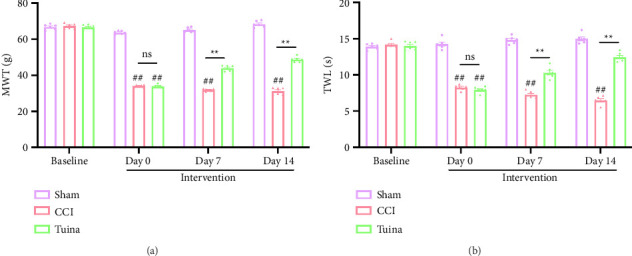
Effect of Tuina on CCI-induced mechanical allodynia and thermal hyperalgesia (*n* = 6). (a) The MWT results for the injured hindpaws of rats. (b) The TWL results for the injured hindpaws of rats. ^##^*p* < 0.01 vs. Sham. ^∗∗^*p* < 0.01 vs. CCI.

As shown in Figure 2, we evaluated the changes in pain thresholds of rats using the MWT and TWL tests. Baseline MWT and TWL values were similar across all groups. On Day 0 of intervention (the 7th day postmodeling), both the CCI and Tuina groups exhibited significantly reduced MWT and TWL values compared with the Sham group (*p* < 0.01), indicating the development of mechanical allodynia and thermal hyperalgesia. On Days 7 and 14 of intervention (i.e., Days 14 and 21 postmodeling), MWT and TWL values in the Tuina group were significantly higher than those in the CCI group (*p* < 0.01). These data indicate that Tuina significantly increases pain thresholds in CCI rats.

### 3.2. Tuina Can Effectively Alleviate the Injury of DRG Neurons in CCI Rats

**Figure 3:**
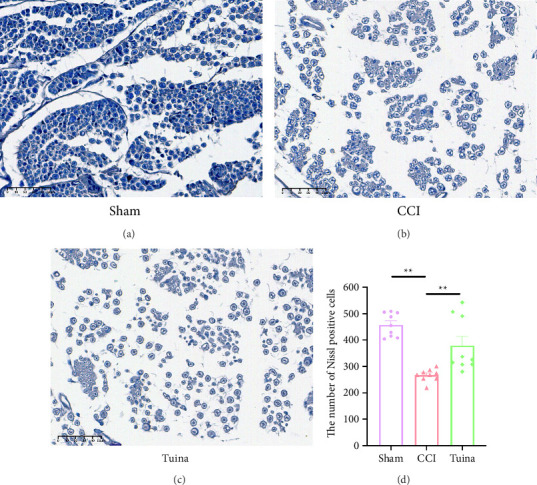
Effect of Tuina on Nissl bodies in DRG neurons of CCI rats (*n* = 3). (a–c) Representative images of Nissl staining in the DRG. Original magnification, ×  400 and (d) the number of Nissl body in the DRG. ^∗∗^*p* < 0.01.

As shown in Figure 3, Nissl staining was employed to observe the damage of DRG neurons in rats. The CCI group showed a significant reduction in the number of Nissl bodies compared to the Sham group (*p* < 0.01), suggesting severe damage to DRG neurons following CCI. The Tuina group showed a significant increase in the number of Nissl bodies compared with the CCI group (*p* < 0.01). These data suggest that Tuina significantly alleviated the loss of DRG neuronal cells in CCI rats.

### 3.3. Tuina Can Significantly Inhibit the Excessive Activation of TRPV4 Ion Channel in the DRG Induced by CCI

**Figure 4:**
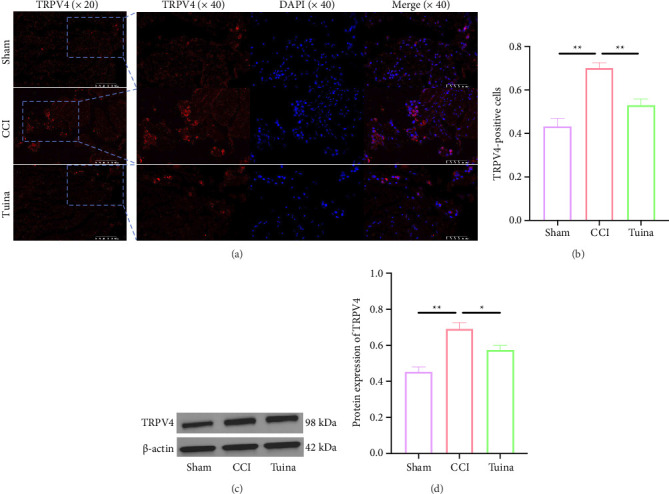
The effect of Tuina on TRPV4 ion channels in the DRG of CCI rats (*n* = 3). (a) Representative images of immunofluorescence of TRPV4 in DRG; (b) immunofluorescence quantification of TRPV4; and (c and d) representative gels and quantification of TRPV4. ^∗^*p* < 0.05, ^∗∗^*p* < 0.01.

As illustrated in Figure 4, we assessed TRPV4 expression in DRG by immunofluorescence (Figures 4(a) and 4(b)) and WB (Figures 4(c) and 4(d)) to investigate the underlying mechanism of Tuina. Compared with the Sham group, the expression of TRPV4 in the CCI group was significantly elevated (*p* < 0.01). In contrast, TRPV4 expression in the Tuina group was significantly reduced compared to the CCI group (*p* < 0.01, *p* < 0.05). These findings suggest that Tuina partially inhibits TRPV4 overactivation in the DRG following CCI.

### 3.4. TRPV4 Channels in the DRG Contribute to the Analgesic Effect of Tuina

**Figure 5:**
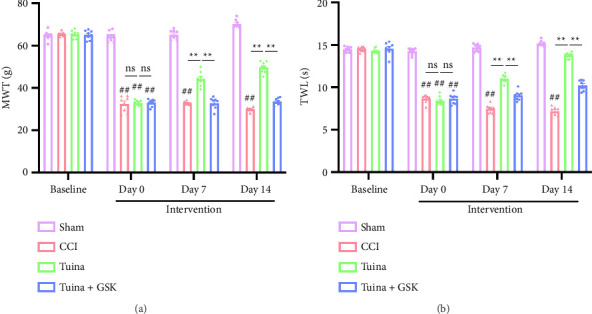
Results of behavioral tests in each group of rats (*n* = 8). (a) Results of MWT in rats and (b) results of TWL in rats. ^##^*p* < 0.01 vs. Sham. ^∗∗^*p* < 0.01.

As shown in Figure 5, to further investigate the role of TRPV4 in Tuina-induced analgesia, the TRPV4 agonist GSK1016790A was administered intraperitoneally into rats 30 min before Tuina treatment, and the changes in their pain threshold were observed. MWT and TWL values in the Tuina + GSK group were significantly lower than those in the Tuina group (*p* < 0.01). These results suggest that application of the TRPV4 agonist GSK1016790A partially reverses the analgesic effect of Tuina.

### 3.5. Tuina Can Alleviate the Excessive Excitation of L4-L6 DRG Neurons in CCI Model Rats

**Figure 6:**
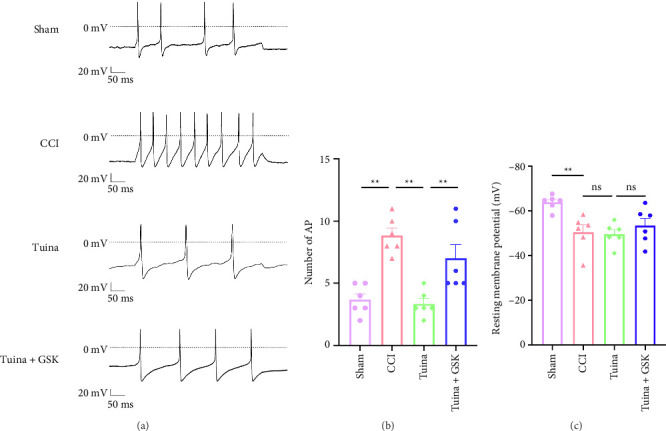
The impact of Tuina on electrophysiological alterations in DRG neurons of CCI rats (*n* = 6-7 cells). (a) Illustration of action potentials in DRG neurons; (b) number of action potentials of neuronal cells in DRG neurons; (c) changes in the resting membrane potential in DRG neurons. ^∗∗^*p* < 0.01.

As shown in Figure 6, we used patch clamp to evaluate the electrophysiological changes of DRG neurons. Compared with the sham group, the CCI group exhibited a significant increase in both the number of action potentials (Figures 6(a) and 6(b)) and the resting membrane potential (Figure 6(c)) in DRG neurons (*p* < 0.01). Compared with the CCI group, the number of AP of DRG neurons significantly decreased after Tuina intervention (*p* < 0.01), while there was no significant difference in the RMP (*p* > 0.05). Compared with the Tuina group, the number of AP of DRG neurons in the Tuina + GSK group significantly increased (*p* < 0.01), indicating that GSK1016790A partially counteracted the effect of Tuina. These data indicate that Tuina mitigated the excessive excitation of DRG neurons in CCI rats, potentially by inhibiting the activation of TRPV4 channels.

### 3.6. Tuina Regulates the CaMKII/CREB Pathway via TRPV4 to Alleviate Inflammation and Play an Analgesic Role

**Figure 7:**
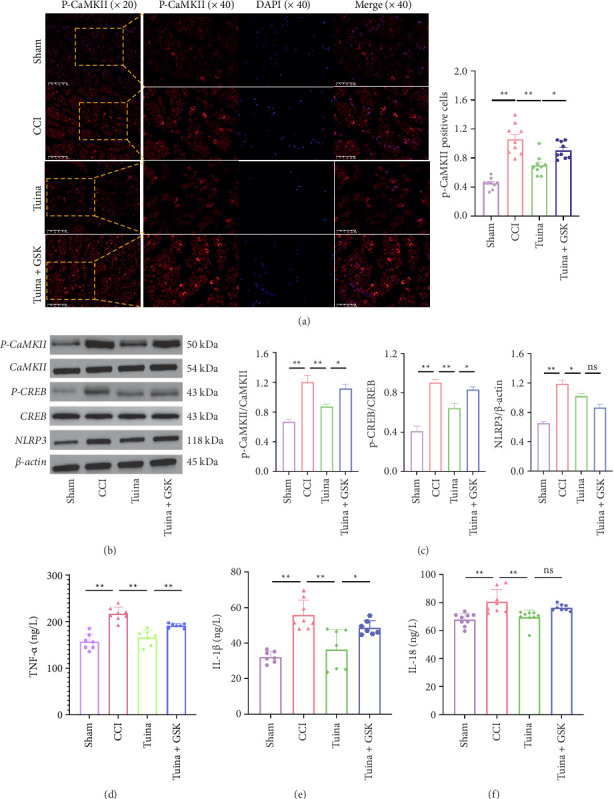
Effects of Tuina on CaMKII/CREB/NLRP3 pathway and proinflammatory cytokines TNF-α, IL-1β, and IL-18 in CCI Rats (*n* = 3). (a) Representative images and quantitative analysis of immunofluorescence of p-CaMKII; (b) representative Western blot bands of P-CaMKII, CaMKII,P-CREB, CREB, and NLRP3 in the each group; (c) quantitative analysis of P-CaMKII/CaMKII, P-CREB/CREB, and NLRP3/β-actin observed in Western blots; and (d–f) secretion of TNF-α, IL-1β, and IL-18 as detected by ELISA. ^∗^*p* < 0.05, ^∗∗^*p* < 0.01.

As shown in Figure 7, compared to the CCI group, the Tuina group exhibited significantly reduced expression levels of p-CaMKII, p-CREB, NLRP3, TNF-α, IL-1β, and IL-18 (*p* < 0.01, *p* < 0.05). Compared to the Tuina group, the Tuina + GSK group showed increased expression of p-CaMKII, p-CREB, TNF-α, and IL-1β (*p* < 0.05) (Figures 7(a), 7(b), 7(c), 7(d), 7(e)). No significant differences were observed in the expression levels of NLRP3 and IL-18 between these two groups (*p* > 0.05) (Figures 7(b), 7(c), 7(f)). These findings suggest that Tuina alleviates pain by modulating the CaMKII/CREB pathway and downregulating the expression of proinflammatory cytokines TNF-α and IL-1β via TRPV4 signaling.

## 4. Discussion

This is a promising study that demonstrates the therapeutic effects of Tuina in CCI rats from a neuroelectrophysiological perspective and highlights its potential as a treatment for NP. In the present study, behavioral pain tests demonstrated that Tuina attenuates CCI-induced mechanical allodynia and thermal hyperalgesia. Tuina improved DRG neuronal injury, suppressed neuronal hyperexcitability, inhibited TRPV4 activation and phosphorylation of CaMKII and CREB, and modulated the local inflammatory microenvironment, thereby exerting both neuroprotective and analgesic effects in the CCI rat model.

In recent years, Tuina has been widely applied in the treatment of peripheral nerve injuries. Tuina exerts analgesic effects by inhibiting microglial activation in the spinal dorsal horn, modulating inflammatory cytokine levels in both the serum and DRG, and regulating the peripheral mechanosensitive ion channel Piezo [29–31]. The three Tuina manipulations—pressing, plucking, and kneading—are warming and relaxing techniques that effectively alleviate pain, numbness, and other neuropathic symptoms. In this study, three acupoints were selected: Yinmen (BL37), Chengshan (BL57), and Yanglingquan (GB34). The deep anatomical layers of Yinmen (BL37) contain the sciatic nerve trunk and the biceps femoris muscle; those of Chengshan (BL57) include the tibial nerve and gastrocnemius muscle; and those of Yanglingquan (GB34) encompass the common peroneal nerve and tibialis anterior muscle [32]. The three manipulations and three acupoints are the combination of the manipulations and acupoints which have been studied by our research group and proved to be effective [33, 34]. In our previous work, we found that MWT and TWL values in rats with sciatic nerve injury were significantly improved after intervention using the three manipulations and three acupoints. Furthermore, we demonstrated that the analgesic mechanism initiated by Tuina was associated with TRP channels [25]. Building upon these findings, this study further explored the peripheral analgesic mechanisms of Tuina.

Peripheral sensitization is considered a core mechanism in both the initiation and maintenance of NP. The DRG, located in the peripheral sensory system, plays a key role in the development and persistence of NP [35]. Following peripheral injury, the structure and function of DRG undergo significant changes. Peripheral injury leads to a reduction in the number of DRG sensory neurons, triggers an immune cascade with inflammatory cell infiltration, and causes upregulation of ion channels and neuropeptides [36, 37]. The infiltration of inflammatory cells contributes to the sustained release of excitatory cytokines, resulting in persistent pain even after the initial injury has resolved [38, 39]. Activation of ion channels leads to excessive neuronal excitation and continuous stimulation of DRG neurons [40]. These changes lead to DRG neuron hyperexcitability, characterized by a reduced threshold and increased spontaneous firing, which in turn transmits large amounts of nociceptive information to the central nervous system in the form of action potentials, culminating in NP [41]. In this study, the CCI model induced pathological changes in DRG neurons and increased their action potential frequency, both of which were ameliorated by Tuina. Therefore, our findings suggest that Tuina can mitigate CCI-induced DRG neuronal damage and suppress neuronal hyperexcitability, thereby alleviating mechanical allodynia and thermal hyperalgesia in CCI rats.

In recent years, the TRPV4 channel has been found to play an important role in the pathogenesis of NP. TRPV4, a mechanosensitive nonselective calcium channel, is widely expressed in the DRG and is associated with mechanosensation and pain perception [7, 8]. Nociceptive stimulation-induced TRPV4 activation following peripheral nerve injury triggers Ca^2+^ influx, thereby enhancing DRG neuron excitability and promoting pain generation [40, 42]. Pharmacological inhibition of TRPV4 not only attenuates pain behaviors but also suppresses NF-κB activation, leading to reduced expression of IL-1β and TNF-α, ultimately mitigating neuroinflammation [43, 44]. Conversely, pharmacological activation of TRPV4 channels promotes the release of Substance P and calcitonin gene-related peptide from spinal afferents, facilitating nociceptive transmission and contributing to mechanical allodynia and hyperalgesia [8]. In the current study, compared to the CCI group, the Tuina intervention significantly reduced TRPV4 expression in the DRG and decreased neuronal excitability, as indicated by reduced action potential frequency. Although several previous studies have reported the neuroprotective effects of Tuina on DRG neurons, none have specifically investigated its impact on neuronal excitability. Thus, our findings provide the first evidence that Tuina alleviates DRG hyperexcitability in CCI rats by inhibiting TRPV4 overexpression.

Per recent studies, TRPV4 functions in a CaMKII-dependent manner. The Ca^2+^ influx mediated by TRPV4 triggers CaMKII autophosphorylation, which subsequently activates the downstream NLRP3 inflammasome and facilitates the secretion of pro-inflammatory cytokines [16]. Pharmacological inhibition of CaMKII significantly reduces pain-related behaviors [45]. CaMKII can also perpetuate peripheral sensitization by activating phosphorylated CREB (p-CREB), thereby inducing both spontaneous and evoked nociceptive hypersensitivity responses [19, 46–48]. In our study, to further elucidate the role of TRPV4 and its downstream signaling pathways in Tuina-induced analgesia, we established a Tuina + GSK (GSK1016790A) group and examined the expression levels of downstream molecules, including p-CaMKII, p-CREB, NLRP3, and the pro-inflammatory cytokines TNF-α, IL-1β, and IL-18. We observed that the expression levels of p-CaMKII, p-CREB, NLRP3, TNF-α, IL-1β, and IL-18 were significantly reduced in the Tuina group compared to the CCI group (*p* < 0.05). However, pharmacological activation of TRPV4 partially reversed the effects of Tuina. These findings suggest that TRPV4 is a key target through which Tuina exerts its analgesic effects. Specifically, Tuina modulates the CaMKII/CREB signaling pathway via TRPV4 regulation, thereby alleviating neuroinflammation and peripheral sensitization and improving NP in CCI rats.

Although this study is the first to provide electrophysiological evidence that Tuina reduces the excitability of DRG neurons and alleviates peripheral sensitization, certain limitations remain. Specifically, this study focused exclusively on peripheral mechanisms and did not investigate the role of central sensitization. It is widely recognized that the development and persistence of NP involve coordinated interactions between peripheral and central sensitization. Future research should incorporate central sensitization mechanisms—including synaptic plasticity in the spinal dorsal horn and higher central nervous system regions, as well as dysfunction in the descending inhibitory system—into multilevel investigations to fully elucidate the analgesic mechanisms of Tuina therapy.

## 5. Conclusion

Tuina can mitigate injury and hyperexcitability of DRG sensory neurons, improve the local inflammatory microenvironment, and alleviate peripheral sensitization, thereby reducing symptoms of mechanical allodynia and thermal hyperalgesia induced by peripheral nerve injury. Notably, TRPV4 serves as a therapeutic target in this process. Our findings support Tuina as a promising alternative therapy for NP.

## Data Availability

The data that support the findings of this study are available from the corresponding author upon reasonable request.
